# Detection of mixed infection level of *Plasmodium falciparum* and *Plasmodium vivax* by SYBR Green I-based real-time PCR in North Gondar, north-west Ethiopia

**DOI:** 10.1186/1475-2875-13-411

**Published:** 2014-10-18

**Authors:** Addimas Tajebe, Gabriel Magoma, Mulugeta Aemero, Francis Kimani

**Affiliations:** Pan African University Institute for Basic Sciences Innovation and Technology JKUAT, P.O. Box 62000–00200, Nairobi, Kenya; University of Gondar, P.O. Box 196, Gondar, Ethiopia; Kenya Medical Research Institute, P.O. Box 54840–00200, Nairobi, Kenya

**Keywords:** Mixed infection, *P. falciparum*, *P. vivax*, Microscopic diagnosis, Real-time PCR

## Abstract

**Background:**

Malaria is caused by five *Plasmodium* species and transmitted by anopheline mosquitoes. It occurs in single and mixed infections. Mixed infection easily leads to misdiagnosis. Accurate detection of malaria species is vital. Therefore, the study was conducted to determine the level of mixed infection and misdiagnosis of malaria species in the study area using SYBR Green I-based real time PCR.

**Methods:**

The study was conducted in seven health centres from North Gondar, north-west Ethiopia. The data of all febrile patients, who attended the outpatient department for malaria diagnosis, from October to December 2013, was recorded. Dried blood spots were prepared from 168 positive samples for molecular re-evaluation. Parasite DNA was extracted using a commercial kit and Plasmodium species were re-evaluated with SYBR Green I-based real time PCR to detect mixed infections and misdiagnosed mono-infections.

**Results:**

Among 7343 patients who were diagnosed for malaria in six study sites within the second quarter of the Ethiopian fiscal year (2013) 1802 (24.54%) were positive for malaria parasite. Out of this, 1,216 (67.48%) *Plasmodium falciparum*, 553 (30.68%) *Plasmodium vivax* and 33 (1.8%) mixed infections of both species were recorded. The result showed high prevalence of *P. falciparum* and *P. vivax*, but very low prevalence of mixed infections. Among 168 samples collected on dried blood spot 7 (4.17%) were *P. vivax*, 158 (94.05%) were *P. falciparum* and 3 (1.80%) were mixed infections of both species. After re-evaluation 10 (5.95%) *P. vivax,* 112 (66.67%) *P. falciparum,* 21 (12.50%) *P. falciparum* + *P. vivax* mixed infection, and 17 (10.12%) *Plasmodium ovale* positive rate was recorded*.* The re-evaluation showed high level of mixed infection, and misdiagnosis of *P. ovale* and *P. vivax*.

**Conclusions:**

The result shows that *P. falciparum* prevalence is higher than *P. vivax* in the study area. The results, obtained from SYBR Green I-based real time PCR, indicated that the diagnosis efficiency of microscopy is very low for species-specific and mixed infection detection. Therefore, real time PCR-based species diagnosis should be applied for clinical diagnosis and quality control purposes in order to prevent the advent of drug resistant strains due to misdiagnosis and mistreatment.

## Background

Malaria, together with other infectious diseases such as tuberculosis and HIV, is an important cause of morbidity and mortality. Malaria is caused by the parasite *Plasmodium*, and is transmitted by anopheline mosquitoes. *Plasmodium* parasite has five species affecting humans. These are *Plasmodium falciparum*, *Plasmodium vivax*, *Plasmodium ovale*, *Plasmodium malariae* and *Plasmodium knowlesi*. These species cause approximately 225 million infections and over 600,000 deaths per year. Among them, *P. falciparum* is the most prevalent and common malaria species worldwide, especially in Africa. It causes the most severe form of the disease and is responsible for over 90% of the malaria patients’ death [1–3].

Clinical diagnosis of Plasmodium species by microscopy is not precise. However, it is still the basis of therapeutic care and plays a key role in the diagnosis of febrile patients in malaria endemic areas. Laboratories in malaria-endemic area needs accurate and precise diagnosis of mono-infection and mixed species infections in order to assure proper treatment decision. This helps to prevent the advent of drug resistant parasite population. Reasonable malaria diagnosis and treatment is essential to avoid non-target effects and to save cost on alternative drugs. Accurate and effective diagnosis is the only way of assuring rational treatment and therapy [4].

Microscopic observation of *P. falciparum* infection is influenced by its parasite density. Parasite density of infections by non-falciparum *Plasmodium* species is usually low compared to *P. falciparum* [5]. Therefore, other Plasmodium species are easily missed, particularly in the absence of symptoms. Moreover, in mixed infections, the background of large numbers of *P. falciparum* parasites makes the observation difficult to differentiate other species [6].

Mixed species infection can not only complicate diagnosis, but also alter the severity and morbidity of the disease. Co-existence of *P. falciparum* and *P. vivax* in a single human host suppress each other. *Plasmodium falciparum* can suppress *P. vivax* parasitaemia by interspecies inhibition. Severity of malaria, in mixed species infection, depends on whether it is a *P. falciparum* or a *P. vivax* super infection. *Plasmodium vivax* super infection over an existing *P. falciparum* infection leads to the rise of *P. falciparum* parasitaemia and causes severe malaria. In contrast, *P. falciparum* super infection over an existing *P. vivax* infection reduces *P. falciparum* parasitaemia. Therefore, it prevents the development of severe malaria [7].

The co-existence of *P. falciparum* and *P. vivax* in Ethiopia and the different levels of effectiveness of the anti-malarial drugs against the malaria parasite species demand administration of the right drug to control morbidity and mortality. In the Ethiopian setting, therefore, the current ongoing efforts to increase access to diagnostic services, including the use of appropriate rapid diagnostic tests (RDTs) are expected to have a significant contribution [8]. In malaria-endemic areas of Ethiopia *P. falciparum* and *P. vivax* mixed infections occur with high frequency [9].

Mixed infection incidence is less than the prevalence of individual species and it is also seasonal. Malaria transmission occurs more in wet season than dry season. The seasonal variation is due to the spread of various mosquito species in wet season. The spread of various mosquitoes in malarious area causes high incidence of both single and mixed infections in wet season than dry season [7]. Relatively high prevalence of individual species occurs in a malarious area where anopheline mosquito population is high, but the prevalence level of mixed infection is usually less compared to single infection prevalence because mixed infection occurs either by simultaneous inoculation of different Plasmodium species at a time or inoculation of Plasmodium species at different times on a single host. Simultaneous inoculation rarely occurs, therefore, the rate of mixed infection is less than the rate of single infection [7].

If mixed-species malaria is misdiagnosed as a single *P. vivax* infection, treatment of *P. vivax* increases *P. falciparum* parasitaemia. Mixed-species infections increase the possibility of anti-malarial drug resistance. Hence, a drug-resistant population of *Plasmodium* parasites will emerge [10]. Therefore, accurate diagnosis or species identification of mixed-species malaria is critical for therapeutic decisions [5, 10]. It helps to manage the selection, dose, and timing of anti-malarial drugs. Mistreatment of a single or multiple species have serious clinical consequences.

Microscopy is a “gold standard” method which remains the most appropriate method for malaria diagnosis in resource-limited area. Most microscopists who see one species might not look for another and misclassification is common due to lack of microscopic skills to differentiate morphological variation within and between species [5, 11]. It is known that microscopy does not reliably distinguish the co-existence of different species [12].

PCR is a better tool for measuring Plasmodium prevalence and detection of both asymptomatic and symptomatic Plasmodium parasites than microscopy. Submicroscopic or low level parasites may not be detected by the conventional microscopy [13]. Molecular diagnostic by real-time PCR offers a more reliable means to detect malaria parasites, particularly at low parasitaemia level and during mixed infections [14, 15]. Thus the purpose of the current study was to re-evaluate the level of single and mixed infection of *P. falciparum* and *P. vivax* using real-time PCR.

## Methods

### Description of the study area

**Figure 1:**
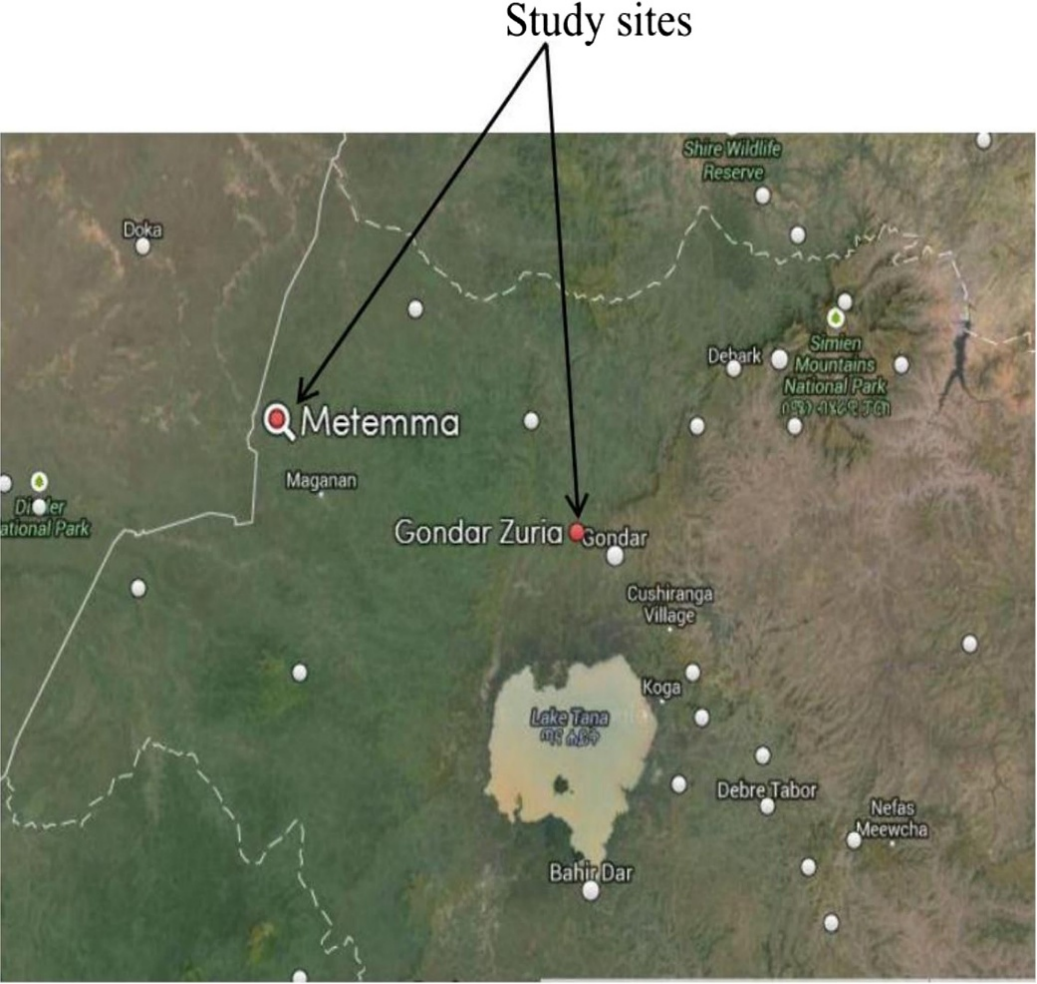
**Map of the study sites.**

The study was conducted in six health centres and one hospital in North Western Gondar, Amhara Regional State, Ethiopia.The health centres are found in Dembia district. The district is located in an altitude range of 1,750 to 2,100 m above sea level; latitude of 12^0^36′N and longitude of 37^0^28′E and 729 km away from the capital Addis Ababa. The district covers an area of 1,270 km^2^ with a total population of about 263,000. The area is endemic to malaria in which *P. falciparum* and *P. vivax* species are commonly reported from microscope diagnosis of febrile patients.The hospital is located in Gendawuha district which is found at a latitude of 12^0^58′N and longitude of 36^0^12′E with an elevation of 685 meter above sea level and approximately 950 km away from the capital Addis Ababa. The area has approximate population of 5,502. The two areas have severe malaria transmission seasons usually October to December (Figure 1).

### Study design

Cross-sectional study was carried out in seven health centres in North Gondar, north-west Ethiopia. This study was conducted during malaria transmission season (October to December, 2013). Six clinics (Tseda, Makisegnit, Chuahit, Kola Diba and Sankisa) and one hospital (Metemma Hospital) were used as a study site to collect samples from outpatients.

### Study participants

Patients who attended the health centres during study period were recruited for the study. Participants who were between six month and seventy years old were included in the study. Infants, under six months of age and pregnant women were excluded. Patients who were reported as *P. falciparum* and/or *P. vivax* positive during health service consultation were asked for consent by health professionals to be part of the study. Finger pricks were done by health professionals for those patients who consented to participate in the study.

### Sampling

After finger pricking, two spots of blood samples were prepared on Whatman 3MM filter paper from each study participant. The blood spots were prepared by dropping three to four blood drops per spot on a piece of filter paper. In total, 168 samples were collected from all study sites (Tseda, n = 14; Makisegnit, n = 14; Sankisa, n = 33; Chuahit, n = 6; Kola Diba, n = 32; Aymba, n = 31 and Metema Hospital, n = 38). From the total samples three samples were mixed infections (Sankisa, n = 2; Kola Diba, n = 1) and seven were reported as *P. vivax* microscopically (Tseda, n = 7). The spots were properly labelled and air dried. Silica gel was added inside each plastic bag to prevent humidity. In order to avoid cross contamination one plastic bag was used for one filter paper. The cards were collected in sealed plastic bag and transported to Kenya Medical Research Institute (KEMRI) for molecular analysis.

### Secondary data

In addition to blood samples collected on filter paper, secondary data was collected from each study site. The data was collected for the second quarter malaria report of the Ethiopian fiscal year (2013) using the report format used by each health centres. This data includes *P. falciparum* and *P. vivax* single infection as well as mixed infection of both species as it was diagnosed by microscopy. During this data collection, the total number of blood film made for malaria microscopic diagnosis, *Plasmodium falciparum* single infection*, P. vivax* single infection and mixed infections of both species were recorded from all the six institutions. Unlike the six health centres, the hospital provided only the total of *P. falciparum* single infection records, although there was no exclusion factor or criteria for the hospital to consider only *P. falciparum* infection. Secondary data was collected in order to determine the prevalence level of *P. falciparum* and *P. vivax* infection in the six health centres as it was diagnosed by microscopy. Due to its severity, *P. falciparum* single infection prevalence level was considered alone in all the study sites.

### Ethical clearance

The research proposal was submitted to the University of Gondar Natural and Computational Science College Ethical Committee for Ethics approval. The Committee Approved the proposed research is Ethical after thorough consideration of the proposed research. Consent form was prepared for study participants. It was explained for participants and parents/guardians in their mother tongue about the purpose of the study before they were being participating in the study. Those who were not consented had withdrawn from participation. In agreement with the clinics and the hospital the cost of drug for treatment of positive patients were covered by the clinics and the hospital with follow up of health professionals.

### Parasite DNA extraction

A piece of dried blood spot of approximately 2 mm-3 mm in size, was cut out with sterile scissors. The pieces were placed in sterile 1.5 ml extraction tubes using flamed forceps. High pure PCR template preparation kit (version 20; Roche diagnostics, GmbH, Germany) was used to extract parasite DNA from dried blood spots (DBS). The extraction was done according to manufacturer’s instruction. The extracted DNA was stored in -20°C freezer until used for PCR.

### Real-time PCR amplification

Two-step PCR was done using AccuPower 2x GreenStar^TM^ qPCR Master mix and Exicycler 96^TM^ (Bioneer South Korea). Exicycler^TM^ version 3.0 Software was used for programming the Exicycler Thermal Block (Exicycler 96) and data analyses.

Consensus primers were used to amplify a species-specific region of the multi-copy 18S rRNA gene. The 18S rRNA gene was used as a target since it contains both highly conserved and variable regions for each of Plasmodium species, and at least five copies of the gene are dispersed on separate chromosomes of the Plasmodium [16]. These consensus species-specific primers amplified both conserved and variable regions of 18S rRNA gene sequences for each of the four Plasmodium species provided that the melting curve analysis step effectively separated the amplicon, obtained from the template DNA for each species, based on nucleotide variations within Plasmodium species. Therefore, the amplification of multi-copy 18S rRNA gene was employed using published primer pair 18S rRNA-F (5′-TAA CGA ACG AGA TCT TAA-3′) and 18S rRNA-R (5′-GTT CCT CTA AGA AGC TTT-3′ which targeted both conserved and variable regions of 18S rRNA gene for detection of Plasmodium at species level [16].

The amplification was done at 50 μl reaction volume. 25 μl master mix mentioned above, 2 μl (10 pmole) forward primer, 2 μl (10 pmole) reverse primer, 16 μl DEPC-distilled (PCR grade) water and 5 μl DNA template were used. Non-template control, known *P. falciparum* laboratory cultured strain 3D7, and microscopically-positive *P. vivax* were used as control group*.* Microscopically *P. vivax* positive samples were reconfirmed before being used as a control, with another PCR assay in which *P. vivax* specific published primer pair Pvr47-F (5′-CTT ATT TTCCGC GTA ACA ATG-3′) and Pvr47-R (5′-CAA ATG TAG CAT AAA AAT CTA AG-3′) were used for conformation [17].

An artificial mix of *P. falciparum* and *P. vivax* DNA were made in order to mimic mixed infection in the following ratios: 100 μl:0 μl, 75 μl:25 μl, 50 μl:50 μl, 25 μl:75 μl, 0 μl:100 μl). The results from these ratios were compared with experimental samples. Thirty-four (20%) of the samples were replicated twice to validate reproducibility of the experiment.

**Figure 2:**
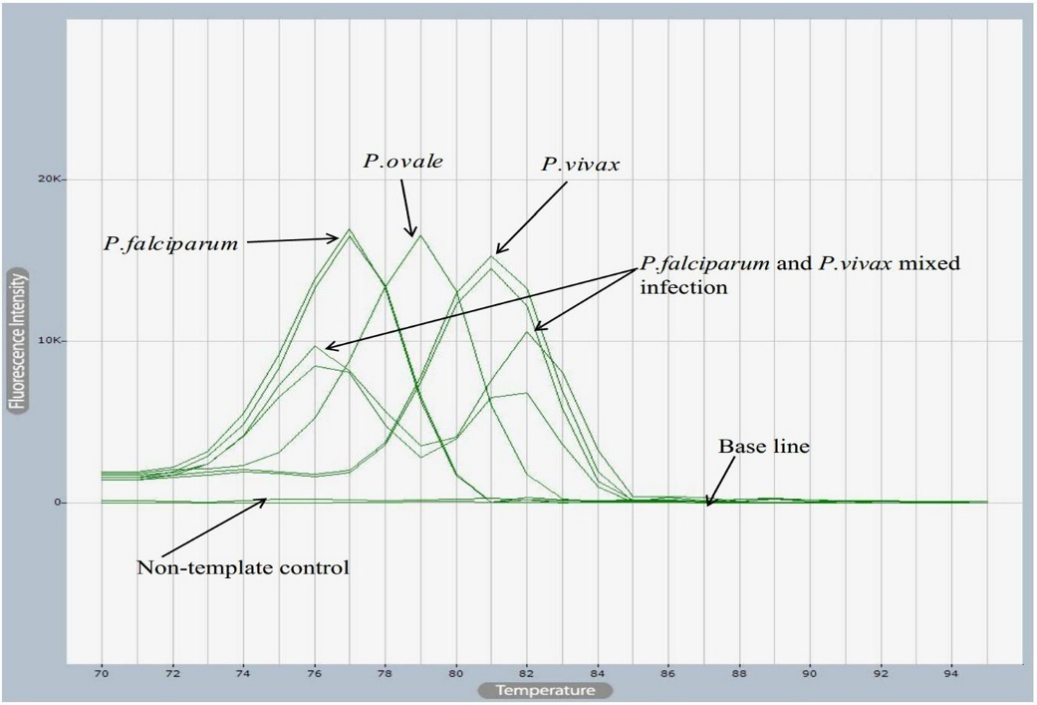
**Melting curves of both single and mixed species infections obtained from SYBR Green I-Based real-time PCR.**

The reaction condition was done as first initiation step at 95°C for 5 minutes twice; denaturation step at 95°C for 20 second; combined annealing and extension at 55°C for 30 second followed by scanning of the amplification product. This two-step PCR was run for continuous 40 cycles. After amplification the product was immediately subjected for melting at 70°C to 94°C, at ramping rate of 1.0°C, for 2 seconds. In order to detect Plasmodium species based on respective temperature profile, the primary differential (F’) was selected from melting curve window. The shape and peaks of the melt curve was carefully observed. The dual and single melting curve peaks were observed from 73°C to 82°C. The most curve peaks were clustered from 77°C +/- 1. The melt curve peaks were shown as discrete variable values. If the curves were observed as dual sharp peaks, more than a difference of 1.5°C, then the peaks were recorded as mixed infections. If the curve was observed as a single sharp peak the result was recorded as mono-infection. *Plasmodium falciparum* infection was represented at 75°C to 78°C temperature range. *Plasmodium vivax* mono-infection was represented at peaks lied on 80°C or above. *Plasmodium ovale* was determined from the experimental set up and recorded at 79°C. This result was confirmed from published paper results which used the same primer pair and experimental set up [16]. If dual peaks at 77°C and 80°C observed then it represented mixed infection of *P. falciparum* and *P. vivax.* In order to confirm both single and mixed infections the known positive controls of *P. falciparum* and *P. vivax* species were included in each experiment. The single or dual melting curve peaks and temperature profiles obtained from the positive controls were compared with melting curve peaks and temperature profiles obtained from the clinical samples (Figure 2).

The real-time PCR results were recorded from amplification curve and melting curve after each run of PCR. The amplification curve in threshold and log scale format was obtained from the Exicycler software in order to observe target gene amplification and the presence of primer dimers, non- specific amplification respectively. The amplification curve in log scale form clearly presents the exclusion of the “noise” or non -specific amplification and primer dimer in the left bottom side of the curve. The default auto-threshold and log scale were used to obtain these curves.

## Results and discussion

**Figure 3:**
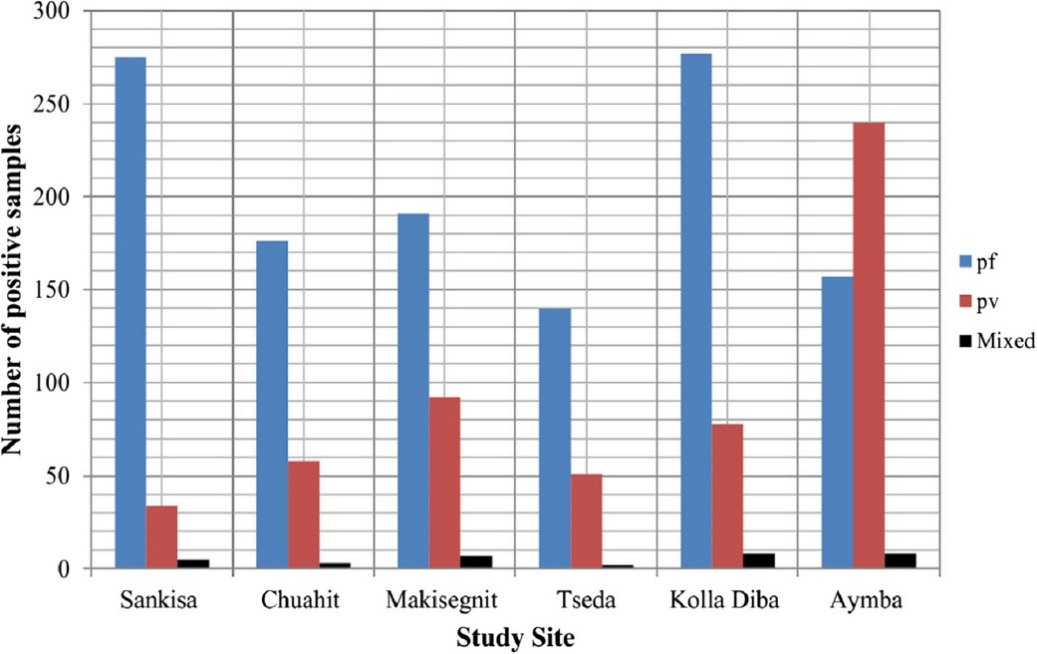
**Microscopic results of single and mixed species infections.**

Out of the 7,343 febrile patients who were attending the outpatient department for malaria diagnosis, 1,802 were reported to have either *P. falciparum* or *P. vivax* single infection or a mixed infection of both species. The report did not include *P. ovale* and *P. malariae* infection. Among these positive cases 1,216 (67.48%) were infected with *P. falciparum* infection, 553 (30.68%) with *P. vivax*, and 33 (1.83%) with both species. According to microscopic diagnosis record, *P. falciparum* and *P. vivax* infection were the most common Plasmodium species with first and second prevalence rate, respectively, in the study area [2] (Figure 3).

Among the 1,802 positive patients, 0.83% were below one year old infants, 6.71% were children between one and four years of age, 12.93% were between five to fourteen years of age, the remaining 79.52% were above fifteen years of age. This report showed that the different exposure rate of infections with regard to age group. Among 1,802 positive patients, 80.85% were males and 19.15% were females. Males were field workers and more infected than females which suggest a higher exposure to mosquito bites. Insecticide-treated bed nets are not commonly used by field workers in the study area, and this could explain the different rate of infection [18].

**Figure 4:**
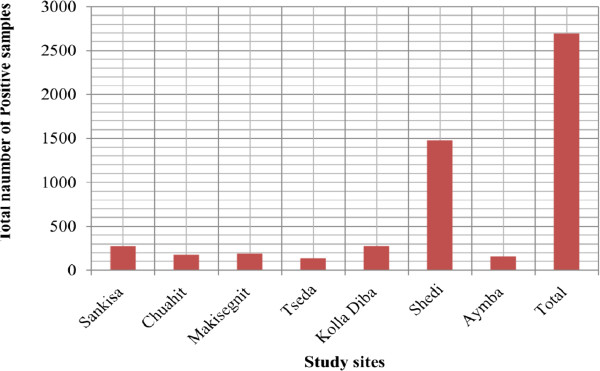
**Prevalence of** ***P. falciparum*** **single infection from microscopy.**

The prevalence of *P. falciparum* mono-infection was considered in all study sites to compare its prevalence in each study site. The prevalence was higher in Metema Hospital than the rest of study sites. The hospital is found in relatively lower land than the other study sites. It provides medication for many people compared to other study sites. In addition, this hospital is found in an investment area in which seasonal labour workers come to the area where they are exposed to malaria infection (Figure 4).

Out of the 168 samples 7 (4.17%) were *P. vivax,* 158 (94.04%) were *P. falciparum,* and three (1.79%) were mixed infection of both species microscopically. The re-evaluation of these 168 samples by SYBR Green I-based real-time PCR method and based on melting curve peaks indicated 112 (66.7%) *P. falciparum*, 10 (5.6%) *P. vivax*, 17 (10.12%) *P. ovale,* and 21 (12.5%) were *P. vivax* + *P. falciparum* mixed infections. Eight (4.8%) were negative for any one of the malaria parasite species. *Plasmodium ovale* was not commonly reported by microscopic diagnosis in the study area. Although *P. ovale wallikeri* and *P. ovale curtisi* have been reported by species-specific nested PCR in Dembia district, which is the same study area [19], they were not distinguished in the present study. The low prevalence of *P. vivax* was not concordant with other studies. Other recent studies showed that *P. vivax* is second highest malaria species in malaria endemic area of Ethiopia [2, 20].

The prevalence of malaria parasite infection obtained from Giemsa stain and its re-evaluation using the SYBR Green I-based real-time PCR method shows a high discrepancy (Table 1). Real-time PCR detection of Plasmodium species is more sensitive than microscopic diagnosis especially when the patient harbours mixed and/or a low parasitaemia [21, 22]. In this study, the SYBR Green I-based real-time PCR assay for Plasmodium species detection is more sensitive than microscopy (Table 1). Real-time PCR detection of Plasmodium species by melting curve analysis is relatively fast and accurate compared to other molecular methods [23]. Melting curve analysis based on nucleotide variations within the amplicon provided a basis for accurate differentiation of Plasmodium species [16]. Therefore, dissociation of PCR product at specific temperature Profile effectively separated PCR amplicon based on their GC-content and product length. As a result, Plasmodium species with varied GC-content accurately discriminated with specific temperature profiles at species level.

**Table 1:** **Microscopic and SYBR Green I-Based Real-time PCR Results**

Parasites	Methods	
	Microscopy 168 samples (100%)	Real-time PCR 168 samples (100%)
*P. falciparum*	158 (94.07%)	112 (66.7%)
*P. ovale*	0 (0%)	17 (10.12%)
*P. vivax*	7 (4.16%)	10 (5.6%)
*P. falciparum* and *P. vivax*	3 (1.8%)	21 (12.5%)
Negative	0 (0%)	8 (4.8%)

In this study, the detection process in both amplification and melting step was approximately 2 hours and 20 minutes and Plasmodium species were detected successfully. Melting curve analysis has an advantage to observe all Plasmodium species simultaneously in a single step. In addition, the method enables closed system data analysis in which it prevents post-PCR contamination of the samples unlike gel electrophoresis.

The use of microscopy for febrile patient diagnosis does not have reliability for effective patient treatment. SYBR Green I-based real-time PCR targeted 18S rRNA gene is sensitive and specific to detect all the four types of Plasmodium species [16, 24]. The results obtained in this study agree with above findings. Therefore, the use of SYBR Green I-based real-time PCR with melting curve analysis step is applicable for effective malaria species diagnosis*.* The misdiagnosis of single or mixed infections leads to mistreatment of patients with inappropriate drugs [20].

## Conclusion

The results indicated that malaria is a public health concern in the study area and there is misdiagnosis of parasite species as single and mixed infection. The discordant or mismatched results obtained from microscopic diagnosis and SYBR Green I-based real-time PCR amplification showed that clinical diagnosis of malaria species and detection of mixed species needs special attention in the study area. Even though real-time PCR based species diagnosis is expensive for clinical application in remote areas, the method is highly advantageous to control and manage prevalence of mixed infection, right representation of single species infection, and detection of emergency of drug resistance strains. Clinical diagnosis quality control strategy is important in malaria endemic areas for prompt treatment and control of the disease.

## Abbreviations

PCR: Polymerase chain reaction
DBS: Dried blood spot.
